# Front Transparent Passivation of CIGS-Based Solar Cells via AZO

**DOI:** 10.3390/molecules27196285

**Published:** 2022-09-23

**Authors:** He Zhang, Fei Qu, Hui Li

**Affiliations:** 1 Institute of Electrical Engineering, Chinese Academy of Sciences, Beijing 100190, China; 2 Institute of Physics, Chinese Academy of Sciences, P.O. Box 603, Beijing 100190, China; 3 School of Physical Sciences, University of Chinese Academy of Sciences, Beijing 100049, China

**Keywords:** photovoltaics, thin film solar cells, passivation, AZO films

## Abstract

We report a novel strategy for the front passivation of solar cells via aluminum-doped zinc oxide (AZO) films in the case of CIGS solar cells, leading to the highest efficiency of 15.07% without alkali metal post treatment and anti−reflective layer. The good passivation of CIGS solar cells via AZO films is attributed to the field passivation simulated by the SCAPS−1D software. The AZO films also exhibit high optical transparency both in visible and near infrared wavelength region, high conductivity, and cost−effective fabrication advantage. Importantly, the AZO films are deposited at room temperature via radio−frequency magnetron sputtering, showing that the AZO films are also applicable to other solar cells such as perovskite solar cells. Our work is of significance for advancing the development of CIGS−based photovoltaics devices by the well front passivation of AZO. The wide application of AZO in other solar cells such as perovskite solar cells and related tandem solar cells may also accelerate the development of these solar cells because of potential passivation of AZO, low deposition temperature, and high optical transparency of AZO.

## 1. Introduction

Photovoltaics (PVs) have attracted intense interest both in scientific and industrial fields to realize carbon neutrality. Among PV devices, thin film solar cells including copper indium gallium (di) selenide (Cu(In_x_Ga_1−x_)Se_2_:CIGS) solar cells are eye-catching because of their prominent advantages of tunable band gap in the region of 1.0–1.7 eV (ideal candidates for perovskite tandem solar cells), high stability, low temperature coefficient of −0.32%/K, easy fabrication both on grid and flexible substrates, low−cost, and so on [1,2,3,4,5]. High power conversion efficiency (PCE) is highly desired for their practical applications, which is also beneficial to cost reduction. For this, tremendous efforts are devoted to the improvement of PCE [6]. As we know, PCE can be effectively improved by simultaneously minimizing the open−circuit voltage loss (*V*_oc,loss_ = *E_g_*/*q* − *V*_oc_) and short−circuit current density (*J*_sc_) loss induced by the parasitic optical absorption [1]. The consensus on *V*_oc,loss_ has been reached, predominantly ascribing it to the non−radiative recombination induced by the bulk and interface defects [7]. Many strategies involving the doping of isoelectronic elements such as Ag and Cd into the CIGS absorbing layer [6,8], and passivation of interface defects [9], have been exploited to passivate the unpopular non−radiative recombination defects in CIGS solar cells. These strategies mainly focus on the passivation of defects located in the CIGS and buffer layers and their interface, which is extremely insufficient because many layers and interfaces exist in the CIGS solar cell having a typical configuration of substrate/Mo/CIGS/buffer layer/high−resistive layer (HRT)/transparent conductive oxide (TCO)/metal electrode/anti−reflective layer [8,9,10]. Identification of the effect of defects located at the HRT/TCO interface remains a big challenge, limiting the front passivation of CIGS solar cells. Apart from the well front passivation for CIGS solar cells, achieving TCOs with high optical transparency both in the visible and near infrared (NIR) regions is a key issue for low parasitic optical absorption [10]. In addition, low resistivity of TCO is of importance for achieving high performance in a CIGS solar cell by reducing the electrical loss.

Herein, aluminum−doped zinc oxide (AZO), the widely applied TCO material in the CIGS solar cell thanks to its wide band gap (*E_g_* = 3.37 eV), high optical transparency, low resistivity, and eco−friendly fabrication [11], is used as the TCO layer in the CIGS solar cell. AZO films are deposited via radio−frequency (RF) magnetron sputtering technique at room temperature (RT). The obtained AZO films show high optical transparency in a wide wavelength region (300–1500 nm), and low resistivity of 5.88 × 10^−3^ Ω·cm. The maximum PCE for CIGS solar cell is 15.07%. The well passivated effect, high optical transparency, and low conductivity of AZO contribute to the high PCE. The AZO passivation comes from the field passivation.

## 2. Experiments and Characterizations

**Figure 1:**
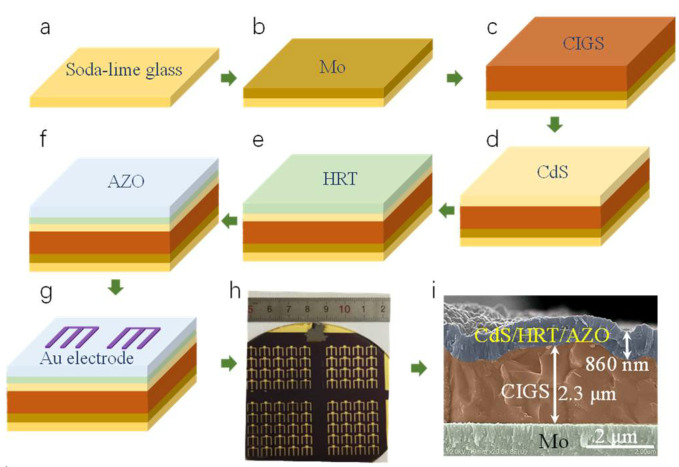
(**a**–**g**) Schematical illustration of the fabrication process for CIGS solar cells. (**h**) Photo images of actual CIGS solar cells. (**i**) Typical cross−sectional SEM image for a CIGS solar cell.

The typical device structure for a CIGS solar cell is: glass/Mo/CIGS/CdS/HRT/AZO/Au, as shown in Figure 1. The Mo hole transport layer is deposited at RT via a one−stage direct−current (DC) magnetron sputtering method with a DC voltage and current of 386 V and 0.65 A, respectively. The CIGS absorbing layer with a thickness of ~2.3 μm (Figure 1i) is grown via a three−stage co−evaporation method in a home−made five−source co−evaporation system, as has been described in our published paper [8]. The CdS buffer layer is synthesized via a chemical bath deposition (CBD) method at 70 °C. The HRT of i-ZnO or ZnMgO is deposited by RF magnetron sputtering technique with a power density of 1.19 W·cm^−2^ and an Ar pressure of 2 Pa. AZO layer with a thickness of ~800 nm (Figure 1i) is grown directly on the top of HRT layer via RF magnetron sputtering technique. The deposition parameters are shown in Table S1, Supporting Information. The Au metal electrode with a thickness of 200 nm is grown by DC magnetron sputtering technique. The fabrication process is systematically illustrated in Figure 1.

The sheet resistance of Mo and AZO is characterized by the four−point probe (Model KYD−1) technique. AZO films are further characterized by the step profile on Dektak Veeco 150 stylus profilometer (Veeco Instrument Inc., Terminal Drive Plainview, New York, NY, USA), X-ray photoelectron spectroscopy (XPS, PHI Quantera), ultraviolet−visible infrared spectrometer (Cary 5000), Hall−Effect measurement (HL 5500 PC), X-ray diffraction (XRD, Bruker D8 Advance), atomic force microscopy (AFM, Bruker Dimension Icon), and scanning electron microscopy (SEM, FESEM, FEI NOVA NANOSEM 450). The current density−voltage (*J*−*V*) curves of CIGS solar cells are collected using a Keithley 2400 source meter under the standard test condition (100 mW·cm^−2^ generated by a Newport Oriel 92193A−1000 solar simulator, 25 °C). External quantum efficiency (EQE) measurements are conducted on the PV Measurement QEX7 equipment. Capacitance-voltage (*C*−*V*) and capacitance-frequency (*C*−*f*) characterizations are performed on the CRX−4K and 4200−SCS at RT. The CIGS solar cell is characterized by the transmission electron microscope (TEM) performed on Tecnai G2 F20 S−Twin. The band diagrams for CIGS solar cells are simulated by SCAPS−1D software. 

## 3. Results

For the application as TCOs on CIGS solar cells, AZO films are deposited at 0.05–0.20 Pa, with a RF power density of 2.38–3.98 W·cm^−2^, a target-substrate distance of 110–150 mm, a substrate temperature of 25 ± 5 °C (RT), 100 ± 5 °C, and 300 ± 5 °C, see Table S1, Supporting Information. 

**Figure 2:**
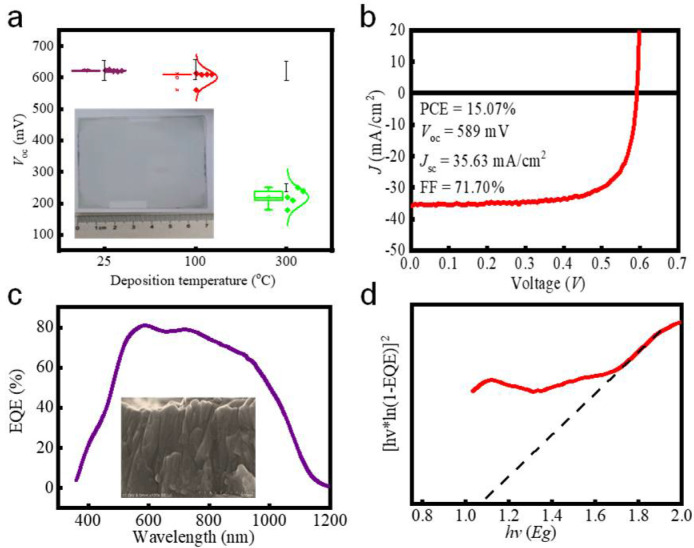
(**a**) The statistical distribution of *V*_oc_ for CIGS solar cells with AZO films as TCOs deposited at different temperatures. The inset shows the photo image of AZO films grown on the soda−lime glass with an area of 100 cm^2^. (**b**) *J*−*V* curve of the CIGS solar cell with the highest PCE of 15.07% free of antireflection layer and alkali metal treatment. (**c**) Typical EQE curve for the CIGS solar cell with an efficiency of 15.07%. The inset shows the column morphology of AZO. (**d**) Band gap of CIGS obtained from the curve of [*hv* × ln(1−EQE)]^2^ vs. *hv*.

As shown in Figure 2a, the performances of CIGS solar cells especially *V*_oc_ are greatly affected by the substrate temperature during the AZO growing process. The *V*_oc_ decreases with the increase in the deposition temperature (25–300 °C), which is mainly ascribed to the junction damage owing to the ion diffusion between CIGS and buffer layer [8]. Consequently, RT deposition of AZO is required to achieve high *V*_oc_ and thus low *V*_oc,loss_ and high performances of CIGS solar cells. Importantly, the value of *V*_oc_ is quite uniform (620–625 mV) when the AZO is grown at RT, indicating the uniformity of AZO films. As a matter of fact, we have grown AZO films with similar performances on the large−scale substrate with an area of 100 cm^2^, see the inset of Figure 2a. Thanks to the high uniformity of AZO, CIGS solar cells are successfully fabricated on a large−scale substrate, displaying high uniformity regarding to *V*_oc_, see Figure 1h. It should be noted that CIGS solar cells are free of Na doping and alkali metal post deposition treatment, which is beneficial to *V*_oc_ [12,13]. The high uniformity of AZO is helpful to the performance uniformity of CIGS solar cells, as shown in Figure S1, Supporting Information. 

The CIGS solar cells also show high *J*_sc_ because of the low parasitic optical absorption and enhanced charge extraction, as determined from the EQE curve (Figure 2c) [14]. Low square resistance of AZO (10.8 Ω/sq deposited at 0.10 Pa) and Mo (0.3 Ω/sq) enhances the charge extraction, see Figure S2 in Supporting Information. The lowest resistivity of 5.88 × 10^−3^ Ω·cm (Figure S3 in Supporting Information) and a square resistance of 10.8 Ω/sq of AZO is due to the Zn_i_ and Vo determined from the XPS results, see Figure S4 in Supporting Information. The average probability of collision (APC), closely related to the mean free path (MFP) and the target−substrate distance (Tables S2 and S3, Supporting Information), of the sputtering ions results in the formation of Zn_i_ and V_o_. The AZO films with good properties are obtained at 0.10 Pa. Importantly, the AZO grown at 0.10 Pa shows column morphology (inset of Figure 2c), consistent with the (002) preferred growth direction determined from the XRD result (Figure S5, Supporting Information). The (002) oriented growth is quite helpful to the current transport. High optical transparency of AZO both in the visible and NIR regions contributes to the low parasitic optical absorption and thus high *J*_sc_ of the CIGS solar cell. As shown in Figure S6b in Supporting Information, the average optical transmittance of AZO is 94.3, 92.2, 86.6, and 85.4% in the 300–900 nm wavelength region grown at 0.10 Pa, a target−substrate distance of 150 mm, and a RF magnetron density of 2.38, 2.78, 3.38, and 3.98 W·cm^−2^, respectively. Importantly, the average optical transmittance in the 900–1500 nm wavelength region is 83.0, 81.4, 82.8, and 80.4% with the RF magnetron density of 2.38, 2.78, 3.38, and 3.98 W·cm^−2^, see Figure S6b in Supporting Information, which are much higher than the reported results (Table S4 in Supporting Information) [15,16,17,18,19,20]. The high NIR transmittance is due to the low carrier density (*n_e_*) of (1.66–1.84) × 10^20^ cm^−3^ as characterized by the Hall−Effect measurement, as shown in Figure S6c in Supporting Information. However, low *n_e_* results in a relatively high resistivity according to: *ρ* = 1/(*qn_e_µ_e_*), see Figure S6c in Supporting Information [1]. The carrier mobility of AZO is 4.25, 5.70, 5.44, and 6.67 cm^2^·V^−1^·s^−1^ with a RF power density of 2.38, 2.78, 3.38, and 3.98 W·cm^−2^ (Figure S6c in Supporting Information), much lower than that (~10.9 cm^2^·V^−1^·s^−1^) of Ti, Ga−doped ZnO [21]. Thus, to simultaneously achieve high optical transparency and low resistivity, high carrier mobility (*µ_e_*) is desired, which can be realized by reducing the impurity and increasing the grain size of AZO [22]. The average grain size of AZO films can be increased by the optimization of the RF deposition power. The average grain size increases from ~36 to ~80 nm when the RF deposition power increases from 2.38 to 3.98 W·cm^−2^. Compact and high crystalline quality of AZO films can be obtained by changing the RF deposition power, see Figure S7, Supporting Information because higher RF power density results in higher energy and momentum of the sputtered atoms, which is helpful to the diffusion of sputtered particles on the substrate and thus the formation of AZO films with compact grains, high crystalline quality, and large grain size. Furthermore, higher RF power density i.e., low substrate−target distance enhances the substrate temperature (Figure S8, Supporting Information), which is also helpful to the high crystalline quality and deep in−diffusion between the high resistive layer and AZO layer and thus large grain size [23]. The surface roughness of the obtained AZO thin films is also increased when the RF power density is 2.38, 2.78, 3.38, and 3.98 W·cm^−2^, respectively, as determined from the three−dimensional AFM image (Figure S9 in Supporting Information). The dependence of roughness on the RF power density further confirms the enlarged grain size and enhanced crystalline quality of AZO films at higher RF power density. However, high RF power density results in the possibility of interfacial damage. Therefore, the RF power density for deposition of AZO for the application in CIGS solar cells is chosen as 2.38 W·cm^−2^ to achieve high optoelectrical properties, high crystalline quality, suitable roughness, and low interfacial damage simultaneously. To further reduce the interfacial damage and achieve superior optoelectrical properties for AZO films, the optimum target−substrate distance is 150 mm. 

The excellent optoelectrical performances of AZO are helpful to the high performances of CIGS solar cells. CIGS solar cells display the highest PCE of 15.07% ± 0.75% with a *V*_oc_ of 589 ± 29 mV, a *J*_sc_ of 35.63 ± 1.78 mA·cm^−2^, and a fill factor (FF) of 71.70 ± 3.58%, see Figure 2b. As shown in Figure 2c, the EQE value decreases at above 1000 nm but still demonstrates a high response at 1200 nm, implying low recombination of photo-generated carriers and thus high interface quality and low interface damage. The obtained band gap for CIGS and AZO is 1.07 eV and 3.47 eV, respectively, calculated from [*hv* × ln(1 − EQE)]^2^~*hv* (Figure 2d) and UV−visible curves (Figure S6d, Supporting Information). Low deposition temperature, low interfacial damage, large−scale uniformity, and high optical transparency render AZO greatly potential applications in CIGS solar cells and other solar cells such as perovskite solar cells, silicon hetero−junction solar cells, and the promising silicon hetero−junction/perovskite tandem solar cells which have no ability in standing for high deposition temperature and high ion bombardment. 

The parasitic optical absorption is further determined from the *J*−*V* curve. The *J*−*V* curve of a solar cells follows the single exponential diode Equation (1),

(1) $$J=J_{0}\exp[\frac{q}{AkT}(V-RJ)]+GV-J_{L}$$

where *J*_0_ is the diode reverse saturation current, *q* is the elemental charge, *A* is the diode ideality factor, *kT* is the thermal energy, and *J_L_* is the photo-generated current. The *R_s_* (series resistance) and *G* (shunt conductance) can be obtained based on the following equations,

(2) $$g(V)=\frac{dJ}{dV}=J_{0}[\frac{q(V-R_{s}\frac{dJ}{dV})}{AkT}]exp[\frac{q(V-R_{s}J)}{AkT}]+G$$

(3) $$r(J)=\frac{dV}{dJ}=\frac{AkT}{q}{\left(J+J_{L}-GV\right)}^{-1}+R_{s}$$

**Figure 3:**
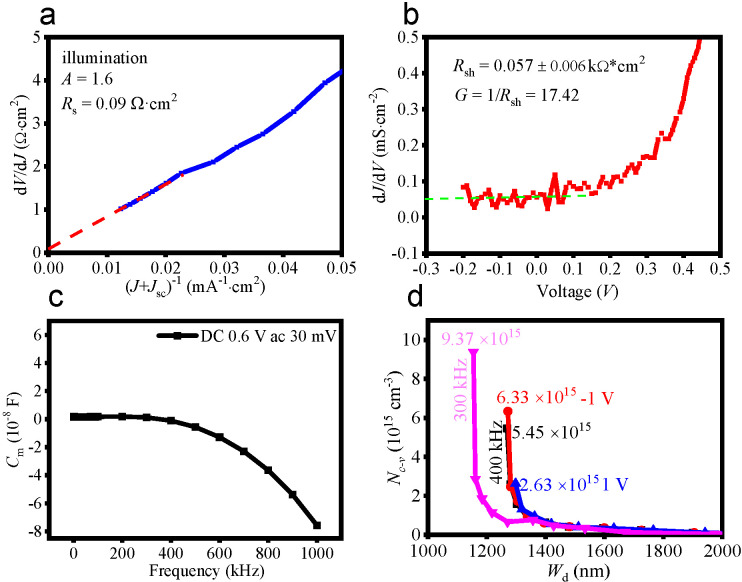
(**a**) *dV/dJ~*(*J + Jsc*)^−1^ curve and the derived *R*_s_ and *A* for the CIGS solar cell. (**b**) *dJ/dV~ V* curve and the derived *R*_sh_ and *G* for the CIGS solar cell. (**c**) *C*_m_−*f* curves for the CIGS solar cell with a −1 V pretreatment and without pre−treatment. (**d**) *N_c-v_*~*W*_dep_ curve sot the CIGS solar cell at 300 and 400 kHz (1 V DC voltage pre−treatment, without a pre−treatment, −1 V DC voltage pre−treatment).

The plot of *r*(*J*)~(*J* + *J*_sc_)^−1^ yields a straight line when *RG* << 1 and *J*_L_ is independent of the voltage, see Figure 3a. The *R*_s_ is as low as 0.09 Ω·cm^2^, which is partly due to the strong carrier conductivity ability of AZO. The obtained *AkT*/*q* from the linear fit of the curve of *r*(*J*)~(*J* + *J*_sc_)^−1^ yields a *A* value of 1.6, indicating a low interface recombination and thereby a high interface quality. The shunt resistance (*R*_sh_) and G (1/*R*_sh_) is 0.057 ± 0.006 kΩ·cm^2^ and 17.42 kΩ^−1^·cm^−2^ derived from the d*J*/d*V*~*V* curve, see Figure 3b. Figure 3c shows the *C*−*f* curve measured on the Keithley 4200−SCS Semiconductor Characterization System with a DC bias of 0.6 V and an AC amplitude of 30 mV. The measured capacitance (*C*_m_) shows little change in the whole frequency region, indicating that *C*_m_ is mainly composed of the frequency−independent junction capacitance (*C*_j_) because the *C*_m_ is comprised of frequency independent *C*_j_ and frequency−dependent trap capacitance (*C*_t_). Thus, the *C*−*f* result further confirms the low interface defects and thus the low interface damage during AZO deposition.

The CIGS solar cell was further characterized via the *C*−*V* measurement. The carrier concentration (*N_c_*_−*v*_) and depletion width (*W*_d_) are derived from the *C*−*V* curves according to the following equations,

(4) $$\begin{array}{l} N_{C-V}=\frac{2}{q\varepsilon_{0}\varepsilon A^{2}[d(1/C^{2})/dV]} \end{array}$$

(5) $$W_{d}=\frac{\varepsilon_{0}\varepsilon A}{C}$$

where *q*, *A*, $\varepsilon_{0},$ ε, *C*, and *V* is the electronic charge (1.60 × 10^−19^ C), device area (0.25 cm^2^), vacuum dielectric constant, dielectric constant of CIGS, junction capacitance, and applied measured voltage, respectively. The built−in field (*V*_bi_) for the CIGS solar cell is obtained from the slope of the *C*^−2^~*V* curve based on the Equation (6),

(6) $$C^{-2}=\frac{2(V_{bi}-V)}{A^{2}\varepsilon_{0}\varepsilon_{r}qN_{C-V}}$$

where *C* is the measured capacitance. From the 1/C^2^~V curve, the *N_c−v_* are obtained, as listed in Table 1. The obtained doping concentration is 9.37 × 10^15^ cm^−3^ (300 kHz) and 5.45 × 10^15^ cm^−3^ (400 kHz), respectively. The largest doping concentration of 9.37 × 10^15^ cm^−3^ is obtained at 300 kHz and the smallest value of 2.63 × 10^15^ cm^−3^ is obtained at 400 kHz with a +1 V DC voltage pre−treatment. The doping concentration decrease at high frequecy is mainly due to the slow change of the carrier. In addition, the carrier concentration is different at forward bias and reverse bias. The forward DC voltage bias only induces the shallow level concentration while the reverse DC voltage bias induces both shallow and deep levels [24]. The little difference between the doping concentration without pre−treatment and reverse DC voltage bias shows low density of deep defects in the CIGS solar cell. The depletion layer width is about ~1.15−1.30 µm, indicating that the obtained CIGS solar cell is not a completely depleted device. The non−complete depleted device results in the increase in the doping concentration at negative voltage pre−treatment and decrease of the doping concentration at forwarding voltage pre−treatment. The obtained *V*_bi_ is 0.42−1.04 V (100 kHz~500 kHz). *N_c−v_*~*W*_dep_ curve (Figure 3d) shows a non−U feature, further indicateing low interface defects [25,26].

**Figure 4:**
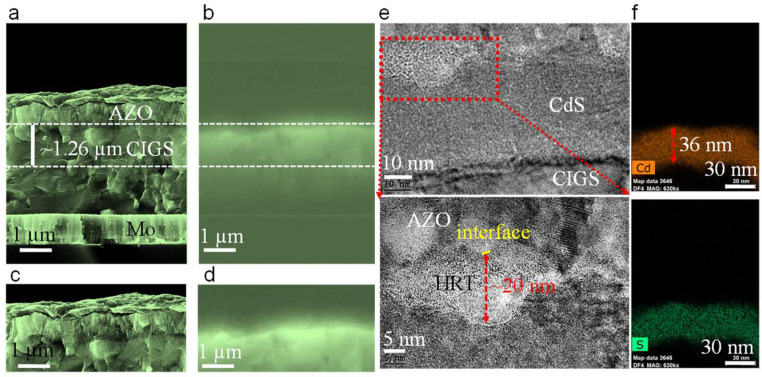
(**a**) Cross−sectional SEM image of CIGS solar cell. (**b**) Corresponding EBIC image of CIGS solar cell. (**c**) Cross−sectional SEM image of CIGS/CdS/i−ZnO/AZO interfaces. (**d**) Corresponding EBIC image of CIGS/CdS/i−ZnO/AZO interfaces. (**e**) High−resolution transmission electron microscopy (HRTEM, upper), and magnified HRTEM (down) images of AZO/high resistive layer (HRT)/CdS in the CIGS solar cell. (**f**) EDX mapping of CdS in CIGS solar cells, indicating the thickness of CdS is about 36 nm.

The depletion layer width is ~1.26 μm determined from the electron beam induced current (EBIC) results, see Figure 4 [27], in good agreement with the *C*−*V* results. The EBIC is predominantly distributed in the depletion region, see Figure 4. Importantly, the current along the interface of Mo/CIGS and CIGS/buffer layer/i−ZnO/AZO is quite high, see Figure 4a–d. The largest current is achieved at the CIGS/buffer layer/i−ZnO/AZO interface. The high current shows the good carrier collection capability along the CIGS/buffer layer/i−ZnO/AZO interface, indicating that the low interfacial defects located at the i−ZnO/AZO interface and thus the high interfacial quality. The high interfacial quality is further confirmed by the high−resolution transmission electron microscopy (TEM) image (Figure 4e). EDX mapping (Figure 4f) of CdS in CIGS solar cells shows the thickness of CdS is about 36 nm.

**Figure 5:**
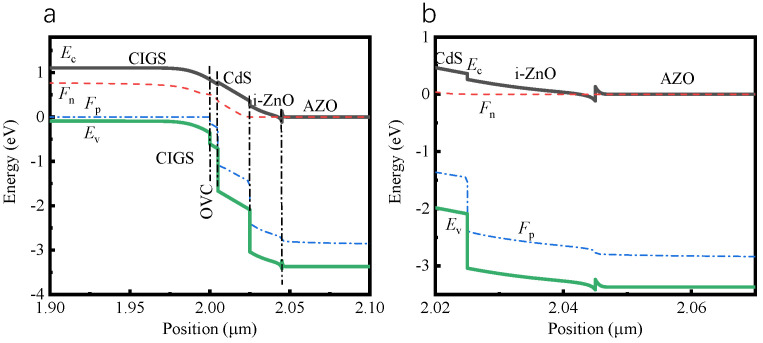
Energy band diagram of (**a**) CIGS solar cell and (**b**) magnified along i−ZnO/AZO in equilibrium under the illustration of AM1.5 G at 300 K simulated via SCAPS−1D software.

The passivation of AZO on the CIGS solar cell is mainly attributed to the field passivation typically induced by the stronger band bending [28]. Figure 5 shows the energy band diagram of CIGS solar cell in equilibrium under the illustration of AM1.5 G at 300 K simulated by SCAPS-1D software with an AZO doping concentration of 10^20^ cm^−3^ (Table S5, Supporting Information). A strong accumulation of majority charge carriers (electrons) at the i−ZnO/AZO interface is determined from the band diagram of CIGS solar cell. However, the minority charge carrier (holes) accumulation at the i−ZnO/AZO interface is greatly suppressed determined by the band diagram of CIGS solar cell. The concentration difference between the majority and minority carriers at the interface reduces the probability of surface recombination. This is also the case for CIGS solar cells by varying the doping concentration (10^20^–10^22^ cm^−3^) of AZO (Figure S10, Supporting Information). 

## 4. Conclusions

In conclusion, AZO films were observed to passivate CIGS solar cells by field passivation, along with the transportation of light and current. The maximum PCE was 15.07% for the CIGS solar cells free of alkali metal post treatment and anti-reflective layer due to the high optical transparency and high conductivity of AZO, and well front passivation by AZO. Our results open a novel strategy for front passivation of CIGS-based solar cells via AZO, which is also potentially applicable to other solar cells such as silicon hetero-junction, perovskite, and related tandem solar cells [4,23,29].

## Figures and Tables

**Table 1:** Doping concentration and depletion layer width for the CIGS solar cell.

Frequency (kHz)	Doping Concentration (10^15^ cm^−3^)	Depletion Layer Width (nm)
300	9.37	1154
400	5.45	1268
400 (−1 V)	6.33	1271
400 (1 V)	2.63	1297

## Data Availability

Not applicable.

## Supplementary Materials

The following supporting information can be downloaded at: https://www.mdpi.com/article/10.3390/molecules27196285/s1, Figure S1: The statistical distribution of (a) PCE, (b) *V*_oc_, (c) *J*_sc_, and (d) FF of CIGS solar cells; Figure S2: The square resistance of AZO films; Figure S3: (a) Dependence of growth rate (thickness/growth time) and resistivity of AZO films grown with a RF power density of 2.38 W·cm^−2^, a target-substrate distance of 150 mm, a substrate temperature of 25 ± 5 °C (RT), and a deposition pressure of 0.05–0.20 Pa. The thickness of AZO is 260, 280, 260, and 250 nm when the deposition pressure is 0.05, 0.10, 0.15, and 0.20 Pa, respectively. (**b**) Relationship between MFP, APC of Al, Zn, and O and deposition pressure; Figure S4: XPS spectrum of (a) AZO, (b) Zn 2*p* core level, (c) Al 2*p* core level, and (d) O 1*s* core level; Figure S5: Typical XRD pattern of the obtained AZO thin film grown at 0.10 Pa; Figure S6: (a) Growth rate of AZO thin films obtained at 2.38–3.98 W·cm^−2^ with a target-substrate distance of 150 mm and a deposition pressure of 0.10 Pa. The thickness of AZO is 280, 305, 420, and 490 nm grown with a RF power density of 2.38, 2.78, 3.37, and 3.98 W·cm^−2^. (b) Transmittance spectra of AZO in the wavelength of 300–1500 nm. (c) Mobility, carrier density, and resistivity of AZO thin films obtained at 2.38–3.98 W·cm^−2^ with a target-substrate distance of 150 mm and a deposition pressure of 0.10 Pa. (d) (*ahv*)^2^ versus photo energy of AZO thin films; Figure S7: SEM images of AZO thin films obtained at RF power density of (a) 2.38 W·cm^−2^, (b) 2.78 W·cm^−2^, (c) 3.38 W·cm^−2^, (d) 3.98 W·cm^−2^ under a deposition pressure of 0.10 Pa and a target-substrate distance of 150 mm; Figure S8: (a) Resistivity of AZO obtained with target-substrate distance of 110 mm, 130 mm, and 150 mm, RF power density of 2.78 W·cm^−2^, and deposition pressure of 0.10 Pa. AFM images of AZO obtained with a RF power density of 2.38 W·cm^−2^, a deposition pressure of 0.10 Pa, and a target-substrate distance of (b) 7 cm and (c) 11 cm; Figure S9: Three-dimensional AFM image of AZO obtained with a RF power density of (a) 2.38 W·cm^−2^, (b) 2.78 W·cm^−2^, (c) 3.38 W·cm^−2^, (d) 3.98·W cm^−2^. (e) Dependence of AZO roughness on the RF power density; Figure S10: Energy band diagram of CIGS solar cell in equilibrium under the illustration of AM1.5 G at 300 K simulated via SCAPS-1D soft-ware by varying the doping concentration in AZO. (a) 1 × e^22^ cm^−3^, (b) 1 × e^21^ cm^−3^.; Table S1: Deposition parameters for AZO films; Table S2: MFP of Al, Zn, O at different pressure; Table S3: APC of Al, Zn, O at different deposition pressure; Table S4: Comparison AZO properties of our AZO films with published results; Table S5: Properties for different layers in a CIGS solar cell for the simulation via SCAPS-1D software.

## Sample Availability

Samples of the solar cells are available from the authors.
