# Supplementary material for: Front Transparent Passivation of CIGS-Based Solar Cells via AZO
Source: Molecules. 2022 Sep 23;27(19):6285. doi: 10.3390/molecules27196285 (PMC9572985; doi:10.3390/molecules27196285)
Supplement: Supplementary file 1 [file molecules-27-06285-s001.zip › molecules-1915417-supplementary.pdf]

Supporting Information

Front transparent passivation of CIGS-based solar cells via AZO

He Zhang,[1] Fei Qu,[1] Hui Li[2,3*]

[1]Institute of Electrical Engineering, Chinese Academy of Sciences, Beijing 100190, China

[2]Institute of Physics, Chinese Academy of Sciences P.O.Box 603, Beijing 100190, China

[3]School of Physical Sciences, University of Chinese Academy of Sciences, Beijing 100049, China

E-mail: lihui2021@iphy.ac.cn

The AZO deposition parameters are listed in Table S1. A batch of AZO films (more than 10 samples) were deposited on different substrates in order to clarify the effect of substrate on the properties of films. For AZO deposition, the sintered AZO ceramic target with a mixture of ZnO (99.99% purity, 98 wt%) and Al_2O_3 (99.99% purity, 2 wt%) was employed as the sputtering target. AZO films were directly grown on glass/Mo/CIGS/CdS/i-ZnO without further treatment. For characterizations, AZO films were also grown on soda-lime glass substrates. Before deposition, the glass substrates were cleaned with acetone, ethanol, and deionized water. After that, the substrates were dried with high purity nitrogen (99.999%, 5N) gas and loaded into the RF magnetron sputtering chamber to grow AZO films. The RF magnetron sputtering chamber was pumped to a base pressure of 5×10^{-4} Pa before AZO deposition.

Table S1. Deposition parameters for AZO films.

Target-substrate distance (mm)	110-150
Sputtering gas	Ar
Ar flow rate (sccm)	15
RF power density (W cm^{-2})	2.38-3.98
Sputtering pressure (Pa)	0.05-0.20
Substrate temperature (°C)	25±5 (RT), 100±5, 200±5

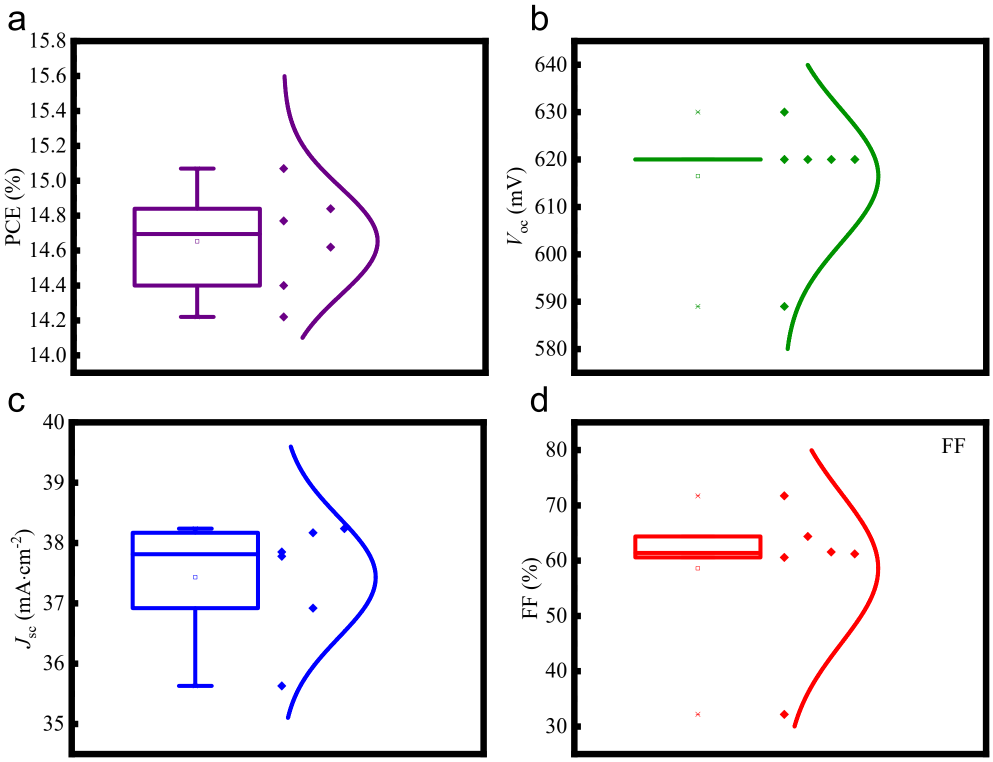

Figure S1. The statistical distribution of (**a**) PCE, (**b**) V_{oc}, (**c**) J_{sc}, and (**d**) FF of CIGS solar cells.

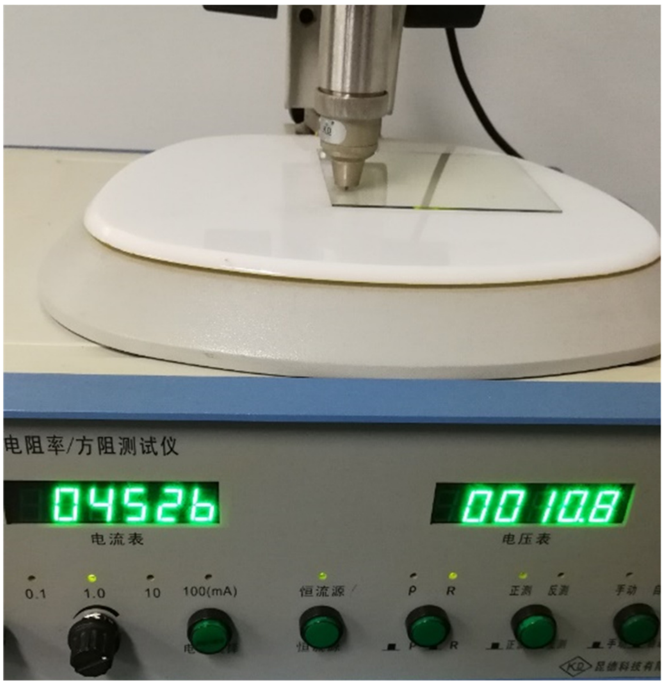

Figure S2. The square resistance of AZO films.

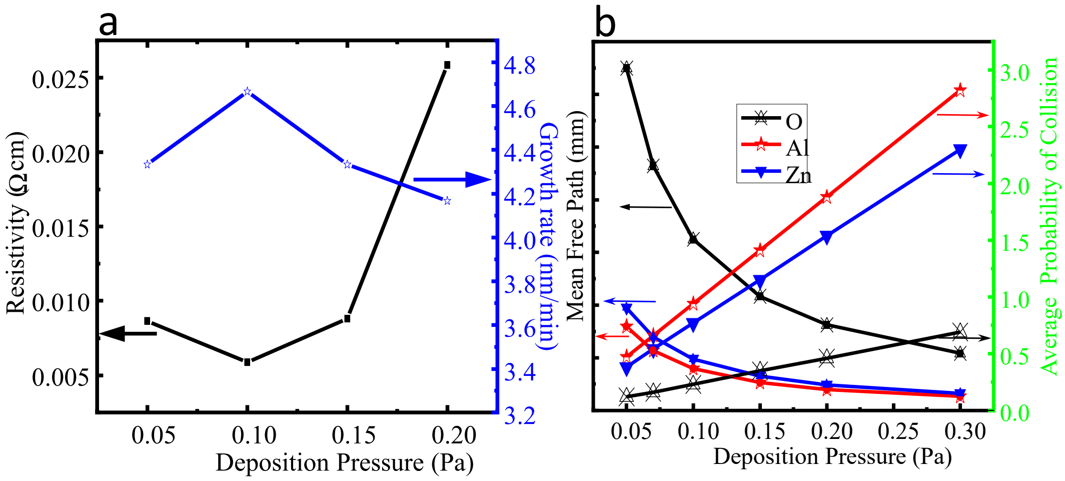

Figure S3. (**a**) Dependence of growth rate (thickness/growth time) and resistivity of AZO films grown with a RF power density of 2.38 W·cm^{-2}, a target-substrate distance of 150 mm, a substrate temperature of 25±5 °C (RT), and a deposition pressure of 0.05-0.20 Pa. The thickness of AZO is 260, 280, 260, and 250 nm when the deposition pressure is 0.05, 0.10, 0.15, and 0.20 Pa, respectively. (**b**) Relationship between MFP, APC of Al, Zn, and O and deposition pressure.

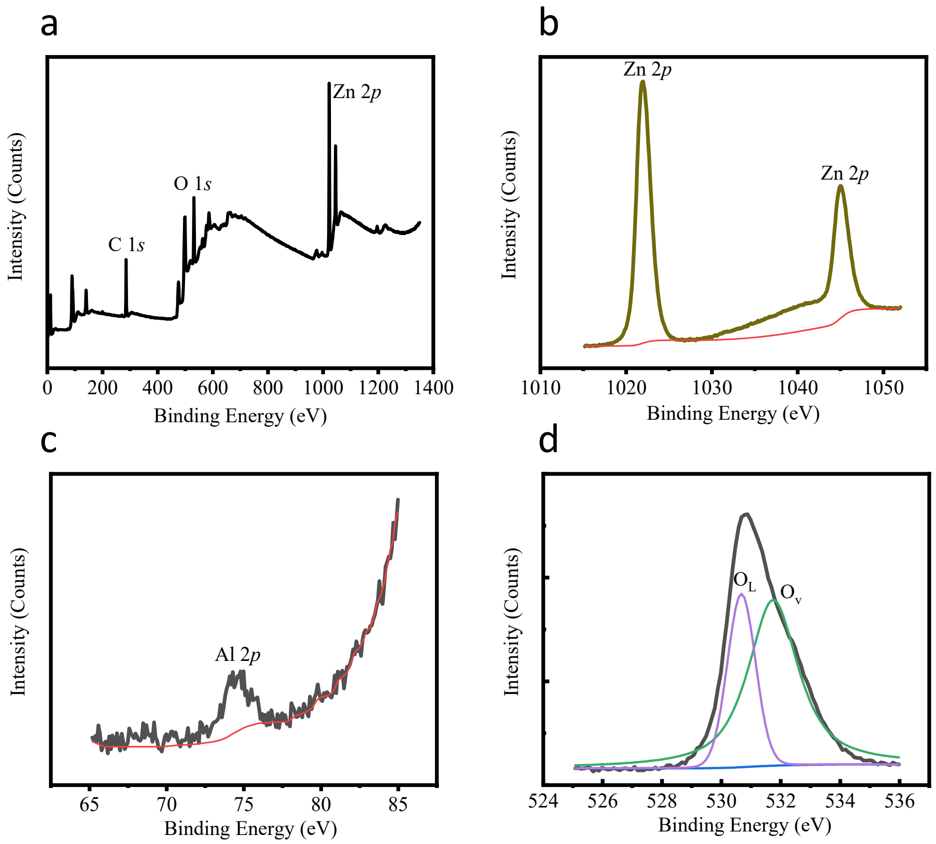

Figure S4. XPS spectrum of (**a**) AZO, (**b**) Zn 2*p* core level, (**c**) Al 2*p* core level, and (**d**) O 1*s* core level.

Table S2. MFP of Al, Zn, O at different pressure.

Atom	MFP (mm) at different Pressure (Pa)			
	0.05	0.10	0.15	0.20
Al	318	159	106	79
Zn	390	195	130	97
O	1299	649	433	324

Table S3. APC of Al, Zn, O at different deposition pressure.

	APC of different atom at different pressure (Pa)			
Atoms	0.05	0.10	0.15	0.20
Al	0.47	0.94	1.41	1.88
Zn	0.38	0.77	1.15	1.54
O	0.12	0.23	0.35	0.46

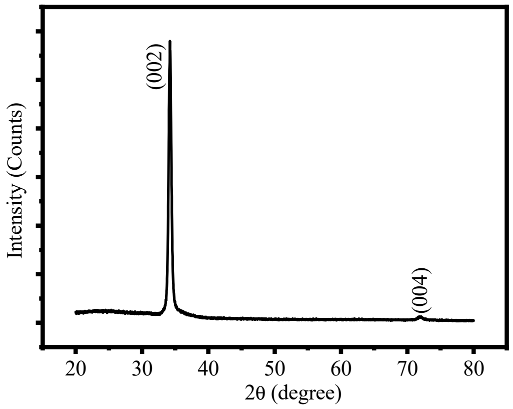

Figure S5. Typical XRD pattern of the obtained AZO thin film grown at 0.10 Pa.

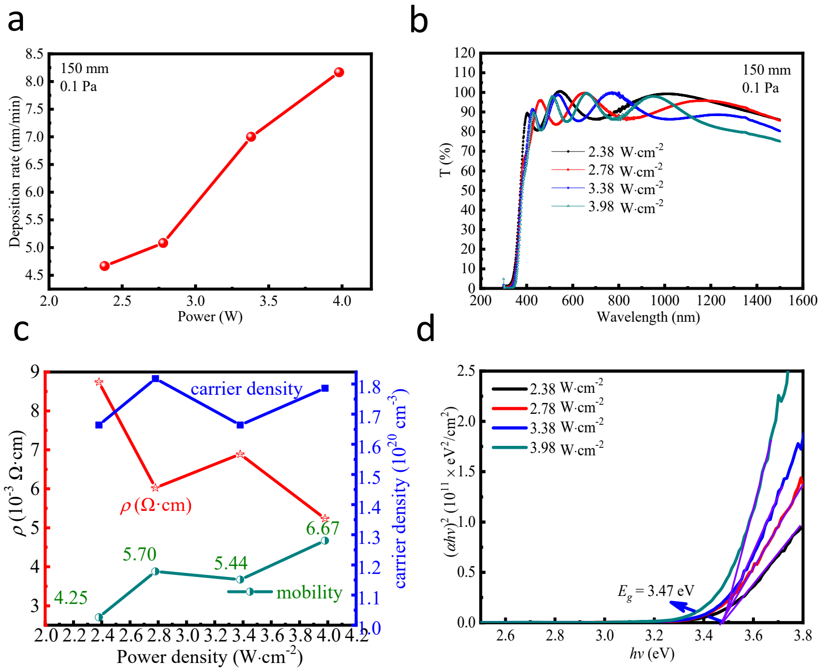

Figure S6. (**a**) Growth rate of AZO thin films obtained at 2.38-3.98 W·cm^{-2} with a target-substrate distance of 150 mm and a deposition pressure of 0.10 Pa. The thickness of AZO is 280, 305, 420, and 490 nm grown with a RF power density of 2.38, 2.78, 3.37, and 3.98 W·cm^{-2}. (**b**) Transmittance spectra of AZO in the wavelength of 300-1500 nm. (**c**) Mobility, carrier density, and resistivity of AZO thin films obtained at 2.38-3.98 W·cm^{-2} with a target-substrate distance of 150 mm and a deposition pressure of 0.10 Pa. (**d**) $(ahv)^2$ versus photo energy of AZO thin films.

Table S4. Comparison AZO properties of our AZO films with published results.

Method	Substrate temperature (ºC)	Transmittance (%) (400-900) nm	Transmittance (%) (900-1500) nm	Resistivity (Ω·cm)	Ref.
RF magnetron sputtering	RT	85		9.80×10^{-2}	1
RF magnetron sputtering	RT	~80		8.16×10^{-4}	2
Pulsed DC magnetron sputtering	RT	~80	~80-55	1.99×10^{-2}	3
MF magnetron sputtering	400 ºC	83		7.56×10^{-4}	4
RF magnetron sputtering	RT			10^{-2}	5
DC magnetron sputtering	150	80		1.78×10^{-3}	6
Reactive RF magnetron sputtering	RT	~80		4.08×10^{-2}	7
**RF magnetron sputtering**	**RT**	**92**	**81**	$\mathbf{2.2 \times 10^{-3}}$	**This work**

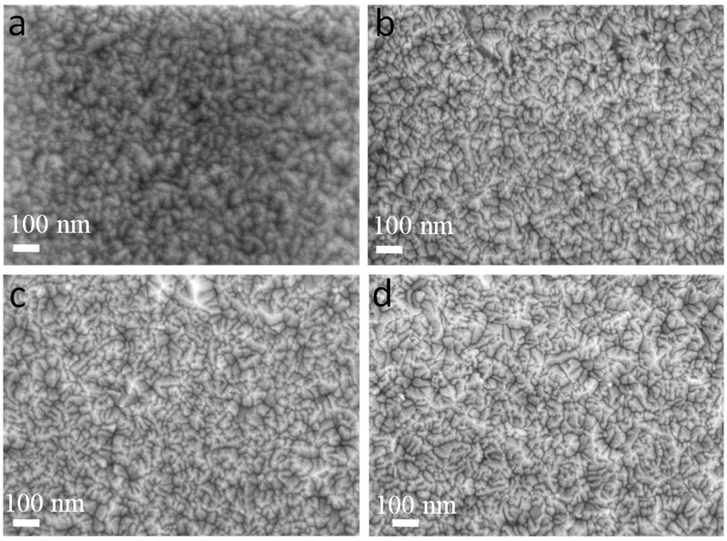

Figure S7. SEM images of AZO thin films obtained at RF power density of (**a**) 2.38 W·cm^{-2}, (**b**) 2.78 W·cm^{-2}, (**c**) W·cm^{-2}, (**d**) 3.98 W·cm^{-2} under a deposition pressure of 0.10 Pa and a target-substrate distance of 150 mm.

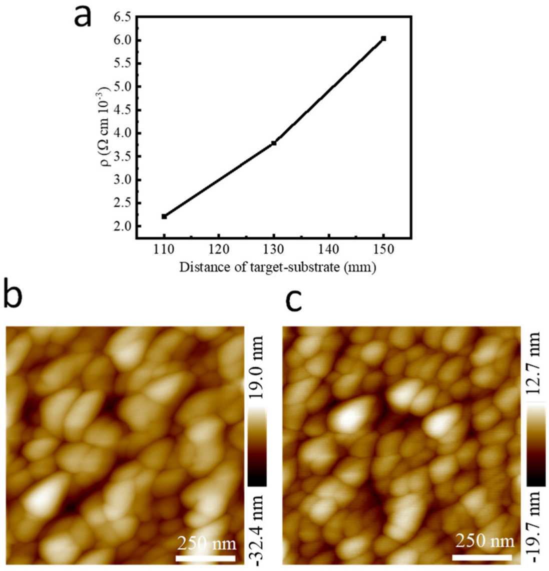

Figure S8. (**a**) Resistivity of AZO obtained with target-substrate distance of 110 mm, 130 mm, and 150 mm, RF power density of 2.78 W·cm^{-2}, and deposition pressure of 0.10 Pa. AFM images of AZO obtained with a RF power density of 2.38 W·cm^{-2}, a deposition pressure of 0.10 Pa, and a target-substrate distance of (**b**) 7 cm and (**c**) 11 cm.

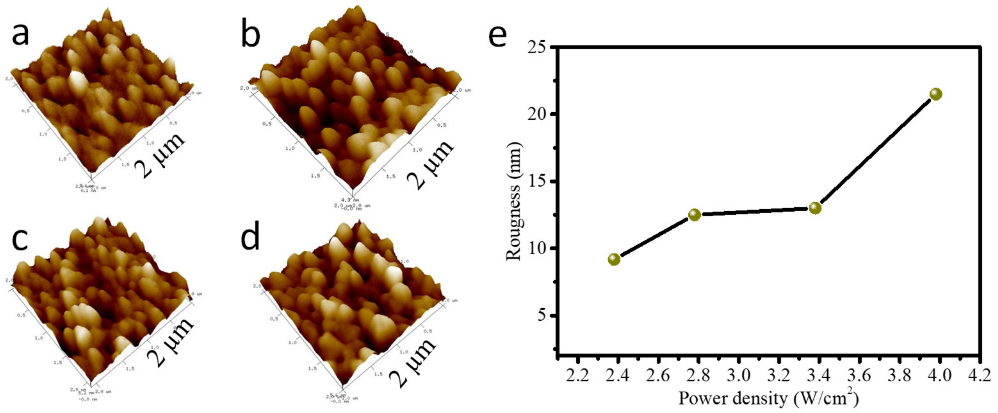

Figure S9. Three-dimensional AFM image of AZO obtained with a RF power density of (**a**) 2.38 W·cm^{-2}, (**b**) 2.78 W·cm^{-2}, (**c**) 3.38 W·cm^{-2}, (**d**) 3.98 W·cm^{-2}. (**e**) Dependence of AZO roughness on the RF power density.

We simulated the CIGS solar cells with the SCAPS-1D software. The device structure of the CIGS solar cell for simulation is: soda lime glass (SLG)/Mo/CIGS/CdS/i-ZnO/AZO/Au. The parameters for different layers in the CIGS are listed in Table S5. The thickness of CdS, i-ZnO, and AZO is 5-100 nm, 20-80 nm, and 200-800 nm. Our simulated results show that little difference is seen in the band diagram of the CIGS solar cells with different thicknesses of CdS, i-ZnO, and AZO. In addition, the effect of the doping concentration of AZO on the band diagram of the CIGS solar cell is also simulated.

Table S5. Properties for different layers in a CIGS solar cell for the simulation via SCAPS-1D software.

Parameters	CIGS	OVC	CdS	i-ZnO	AZO
Thickness (nm)	2000	15	5-100	20-80	200-800
Band gap E_g (eV)	1.0-1.2	1.45	2.45	3.30	3.37
Electron affinity χ (eV)	4.6	4.5	4.4	4.3	4.3
Relative dielectric permittivity ε_r	13.6	10.0	10.0	9.0	9.0
Effective conduction band density N_c (cm^{-3})	$6.8 \times e^{17}$	$2 \times e^{18}$	$1.3 \times e^{18}$	$3 \times e^{18}$	$1 \times e^{20}$
Effective valence band density N_v (cm^{-3})	$1.5 \times e^{19}$	$2 \times e^{18}$	$9.1 \times e^{18}$	$1.7 \times e^{19}$	$3 \times e^{18}$
Electron thermal velocity v_n (cm/s)	$1 \times e^{7}$	$1 \times e^{7}$	$3.1 \times e^{7}$	$1 \times e^{7}$	$1 \times e^{7}$
Hole thermal velocity v_p (cm/s)	$1 \times e^{7}$	$1 \times e^{7}$	$1 \times e^{7}$	$1 \times e^{7}$	$1 \times e^{7}$
Electron mobility μ_n (cm^2/ Vs)	100	100	72	100	100
Hole mobility μ_p (cm^2/ Vs)	12.5	20	20	31	31
Donor concentration N_D (cm^{-3})	0	0	$5 \times e^{17}$	$1 \times e^{17}$	$1 \times e^{20}$ – $1 \times e^{22}$
Acceptor concentration N_A (cm^{-3})	$2 \times e^{16}$	$1 \times e^{13}$	0	0	0

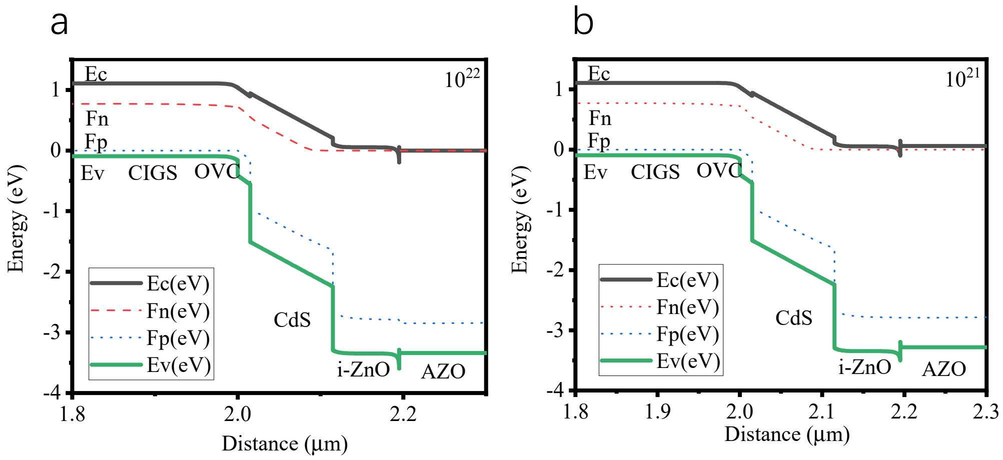

Figure S10. Energy band diagram of CIGS solar cell in equilibrium under the illustration of AM1.5 G at 300 K simulated via SCAPS-1D soft-ware by varying the doping concentration in AZO. (a) $1 \times e^{22}$ cm^{-3}, (b) $1 \times e^{21}$ cm^{-3}.

References：

1 Jeong, S.H.; Boo, J.H. Influence of target-to-substrate distance on the properties of AZO films grown by RF magnetron sputtering. *Thin Solid Films* **2004,** *447-448*, 105-110.

2. Shi, Q.; Zhou, K.; Dai, M.; Hou, H.; Lin, S.; Wei, C.; Hu, F. Room temperature preparation of high performance AZO films by MF sputtering. *Ceram. Int.* **2013**, *39*, 1135-1141.

3. Sarma, B.; Barman, D.; Sarma, B.K. AZO (Al:ZnO) thin films with high figure of merit as stable indium free transparent conducting oxide. *Appl. Surf. Sci.* **2019**, *479*, 786-795.

4. Qian, S.; Kesong, Z.; et al. Room temperature preparation of high performance AZO films by MF sputtering. *Ceram. Int.* **2013**, *39 (2)*, 1135-1141.

5. Hyung Jun, C.; Sung Uk, L.; et al. The effect of annealing on Al-doped ZnO films deposited by RF magnetron sputtering method for transparent electrodes, *Thin Solid Films* **2010**, *518*, 2941-2944.

6. Yanping, X.; Peihong, W.; et al. Deposition and characterization of AZO thin films on flexible glass substrates using DC magnetron sputtering technique. *Ceram. Int.* **2017**, *43*, 4536-4544.

7. Kim, T. W.; Choo, D.C.; No, Y.S.; Choi, W.K.; Choi, E.H. High work function of Al-doped zinc-oxide thin films as transparent conductive anodes in organic light-emitting devices. *Appl. Surf. Sci.* **2006**, *253*, 1917-1920.
